# 4508 Contextual Predictors of Hospitalization and Quality of Life Among Patients on Hemodialysis

**DOI:** 10.1017/cts.2020.264

**Published:** 2020-07-29

**Authors:** Kathryn Taylor, Deidra Crews, Patricia Davidson

**Affiliations:** 1 Johns Hopkins University School of Medicine; 2 Johns Hopkins School of Nursing

## Abstract

OBJECTIVES/GOALS: People engaging in high-risk substance use or experiencing food insecurity or housing instability are at increased risk to develop end-stage kidney disease. This study will examine associations between these risk factors, patient indicators of socioeconomic position, and hospitalization rates and quality of life after initiation of hemodialysis. METHODS/STUDY POPULATION: The proposed study will leverage a prospective cohort design. We will enroll a convenience sample of 330 participants from the same large dialysis organization. Participants will complete measures of socioeconomic position (age, gender, race, ethnicity, education, income, occupation and community poverty); substance use; food insecurity; housing instability; and quality of life at baseline. We will follow participants for 6 months and extract hospitalization counts from the dialysis facility medical record. RESULTS/ANTICIPATED RESULTS: We will generate risk scores (low, medium, high) from measures of substance use, food insecurity and housing instability. We will conduct multiple logistic regression to generate odds ratios comparing risk group membership by indicators of socioeconomic position. We anticipate that low or medium-risk groups will differ from high risk groups by indicators of socioeconomic position. We will conduct Poisson regression to generate incidence rate ratios for 6-month hospitalization rates comparing low or medium-risk and high-risk groups. Lastly, we will conduct multiple linear regression to generate beta coefficients for changes in quality of life scores comparing low or medium-risk and high-risk groups. We anticipate that high-risk groups will have higher hospitalization rates and lower quality of life scores. DISCUSSION/SIGNIFICANCE OF IMPACT: As the prevalence of end-stage kidney disease continues to increase, there is a need for tertiary prevention interventions that reduce costly inpatient utilization and improve health-related quality of life. The proposed study will lay groundwork for the development of interventions to improve patient outcomes and reduce Medicare spending.

